# *In Vitro* Evaluation of Flame-Made Calcium Phosphate Nanoparticles for Antigen Delivery and Immunostimulation

**DOI:** 10.1021/acsanm.5c01535

**Published:** 2025-05-27

**Authors:** Anshika Maheshwari, Rebecca Dookie, Meztlli O. Gaytán, Birgitta Henriques-Normark, Georgios A. Sotiriou

**Affiliations:** † Department of Microbiology, Tumor and Cell Biology, 27106 Karolinska Institutet, Stockholm 171 77, Sweden; ‡ Clinical Microbiology, Karolinska University Hospital, Stockholm 171 77, Sweden; § Department of Chemistry, Science for Life Laboratory, Stockholm University, Stockholm 114 18, Sweden

**Keywords:** nanoparticles, flame spray pyrolysis, ovalbumin, immune potentiators, adjuvant, vaccine

## Abstract

The use of nanoparticles in vaccine formulations has become increasingly prevalent, with the rise of subunit vaccines. However, the production of nanoparticles is often not scalable, presenting a significant challenge for large-scale vaccine manufacturing. Additionally, these nanoparticle delivery systems often require additional immunopotentiators to elicit a robust immune response, further complicating vaccine formulation. In this study, we explore the potential of flame-synthesized calcium phosphate (CaP) nanoparticles, produced via flame spray pyrolysis (FSP) (a highly reproducible and scalable method), as vaccine adjuvants capable of both antigen delivery and immunostimulation. We produced three different CaP nanoparticles with controlled crystallinity and size to screen for their immunostimulatory properties and evaluated their capacity to load and protect the model antigen ovalbumin (OVA) from enzymatic degradation. Our results show that all three CaP nanoparticles significantly enhance antigen internalization and processing by bone marrow-derived dendritic cells (BMDCs), critical for effective T cell activation. OVA conjugated with amorphous CaP nanoparticles outperformed crystalline CaP in increasing the expression of costimulatory markers CD86 and CD80 on BMDCs, as well as enhancing IL-6 production, indicating their potential as effective immunopotentiators. This dual functionality, in addition to the facile synthesis process, could simplify vaccine formulations by obviating the need for separate immunostimulatory agents. This work lays the foundation for further research to establish the flame-made CaP nanoparticle effectiveness and safety as adjuvants *in vivo*.

## Graphical abstract

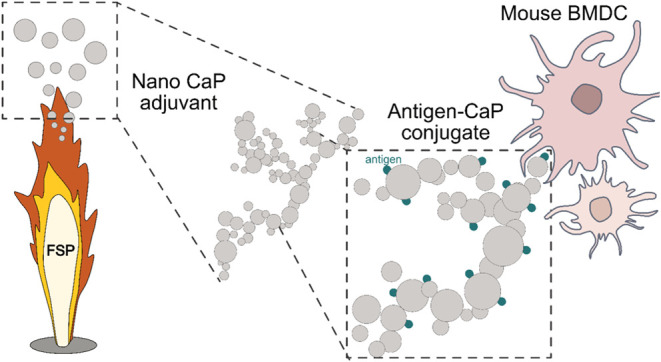

## Introduction

Vaccine adjuvants are critical components in modern vaccinology, serving as catalysts to enhance the immune response to an antigen. Derived from the Latin “adjuvare” meaning “to help”, adjuvants are substances that, when used in conjunction with a specific antigen, produce a more robust immune response than the antigen alone.^1–3^ Adjuvants are highly important; they not only increase the immunogenicity of weak antigens but also enhance the speed and duration of the immune response, stimulate both humoral and cell-mediated immunity, and can reduce the dose of antigen required, thereby decreasing costs and the need for booster shots.^2,4^ For decades, the standard adjuvant used has been “alum”, an aluminum salt-based adjuvant known for its ability to induce a strong antibody response.^1,5,6^ This adjuvant primarily exhibited a depot effect, characterized by a gradual release of antigens, enabling prolonged interaction with immune cells.^2,6–8^ However, as our understanding of the immune system has deepened, new adjuvants have been developed in recent years to target more specific immune responses. These include adjuvants that can stimulate the innate immune system through, for instance, toll-like receptors (TLRs), leading to a more tailored and effective vaccine.^1,9^

Calcium phosphate (CaP) has been employed as an adjuvant due to its natural presence in the human body, making it highly biocompatible and safe for use in vaccines.^10–13^ Its bioresorbable properties in acidic conditions enable gradual absorption and elimination from the body, minimizing long-term risks.^13–15^ The development of the nanoparticulate form of CaP offers several advantages over traditional forms, including controlled particle size, high specific surface area, and pH responsiveness. These features are crucial for enhancing antigen adsorption, protecting against degradation, ensuring controlled antigen release, and eliciting a prolonged immune response.^16,17^ CaP nanoparticles have been shown to induce a robust Th1/Th2 balanced immune response, highlighting its capacity to be an effective vaccine adjuvant.^6,12,18^ In addition, CaP nanoparticles have been associated with favorable IgG responses and a reduced IgE response, indicating their potential to minimize allergic reactions while promoting strong and long-lasting immunity.^13,19–21^ They also have the potential to be tailored for specific immunological effects due to their size, shape, and surface charge.^22^ Currently, CaP nanoparticles are being used in various vaccine formulations, cancer immunotherapy, allergen sensitization, and infectious diseases.^18,19^

Despite the benefits associated with the employment of CaP as adjuvants, the widespread adoption of CaP nanoparticles has been hindered by the challenges associated with their large-scale and reproducible production.^23,24^ Current methods are often inefficient with minimal control over the synthesis of different CaP polymorphs and their crystallinity.^13,16,25^ Achieving minimal batch-to-batch variation for the desired crystal phase while upscaling production is essential for consistent vaccine efficacy and safety, yet this remains a significant obstacle in the nanomanufacturing process.^26–29^ Addressing these production challenges is vital for broader utilization of CaP nanoparticles in vaccine development.

Flame nanoparticle synthesis, particularly flame spray pyrolysis (FSP), has emerged as a promising solution to the challenges of nanomanufacturing, offering a scalable and reproducible method for CaP nanoparticle production.^30–32^ This scalable process has been utilized to create CaP nanoparticles with high drug-loading capacities, exemplified by the formulation of nanoparticles loaded with the LL-37 antimicrobial peptide.^33^ Nonetheless, flame-made CaP nanoparticles have never been utilized as adjuvants in vaccine delivery so far. Given the polymorphic nature of CaP, which can exist in several crystal phases and sizes, it is crucial to systematically study these properties to optimize the efficacy and safety of CaP nanoparticles in biomedical applications and to ensure that the desired biological responses are achieved while minimizing potential side effects.

Here, we perform flame synthesis of CaP nanoparticles, achieving control over their crystallinity and size. We further study their capacity to load the model antigen ovalbumin (OVA) and protect it from enzymatic degradation as well as assist in its internalization and processing by murine bone marrow-derived dendritic cells (BMDCs). The immunomodulatory properties of these CaP nanoparticles were also investigated in detail, providing fundamental insights on the effects of CaP size and crystallinity.

## Experimental Section

### Calcium Phosphate Nanoparticle Synthesis

Flame spray pyrolysis (FSP) was employed for the synthesis of three differently sized calcium phosphate nanoparticles with protocol adapted from Mädler et al.^34^ and Merkl et al.^32^ The liquid precursor was prepared by dissolving calcium acetate hydrate (≥99%, Sigma-Aldrich) in a 1:1 proportion of propionic acid (≥99.5%, Sigma-Aldrich) and 2-ethylhexanoic acid (99%, Sigma-Aldrich). Tributyl phosphate (≥99%, Sigma-Aldrich) was added as a phosphorus source to achieve a Ca-to-P ratio of 2.19. The solution was prepared with total metal (Ca + P) concentration of 0.4 M for L CaP and M CaP particles, and to achieve small particle morphology, it was changed to 0.1 M. The mixture was heated at 70 °C under reflux conditions for 30 min to obtain a clear solution. These liquid precursors were introduced into the FSP nozzle through a capillary using a 100 mL syringe and a syringe pump (New Era Pump Systems, Inc.). O_2_ gas (>99.5%, AGA Gas AB) facilitated the atomization of the precursor solution, subsequently ignited by a methane/oxygen (>99.5%, AGA Gas AB) flamelet. A constant flow rate of 1.5 and 3.2 L/min was maintained for methane and oxygen, respectively, for the premixing pilot flame. The ratio (p/d) between the precursor flow rate (mL/min) and the dispersion oxygen flow rate (L/min) was systematically adjusted to achieve the three different particle sizes. The p/d ratio of 3/8, 5/5, and 10/5 were employed to generate small, medium, and large particles, respectively. The resulting NPs from the process were collected on a glass fiber filter (Hahnemühle FineArt, GmbH) with the assistance of a Mink MM 1144 BV vacuum pump (Busch).

### Nanoparticle Characterization

The morphology characterization of the nanoparticles was performed by using a Talos 120C transmission electron microscope with a 120 kV beam voltage. Carbon-coated copper grids (S160-4, Agar Scientific) were used to deposit the nanoparticle suspension.

The evaluation of the specific surface area (SSA) for the nanoparticles (NPs) was carried out using nitrogen adsorption measurements with a TriStar II PLUS instrument (Micromeritics). Initially, the nanoparticles underwent a 3 h degassing process at 110 °C. Subsequently, N_2_ adsorption took place at 77 K, and the volume of adsorbed gas was quantified. The SSA was determined from the nitrogen adsorption isotherm utilizing the Brunauer–Emmett–Teller (BET) method. Assuming the particles to be spherical with homogeneous density, the primary nanoparticle size was then calculated based on the SSA.

Crystal phase identification of the synthesized nanoparticles was conducted through X-ray diffraction using a Rigaku MiniFlex instrument. The measurements were carried out on the Calcium Phosphate nanoparticle powder at ambient temperature, utilizing 1.5406 Å Cu Kα1 radiation. Diffraction patterns were recorded with a step size of 0.01° in the 2Θ range between 10 and 90°. To determine the crystal phases present, a search against the crystallography open database was performed using Rigaku PDXL software.

The hydrodynamic size and zeta potential of the nanoparticles were determined via dynamic light scattering (DLS) and electrophoretic light scattering (ELS) using the Malvern Zetasizer Ultra. The particles were sonicated in a water-cooled cup horn system (Sonics Vibra-Cell) to disperse the nanoparticle powder in a ultrapure water suspension (1 mg/mL). The measurement was carried out at a particle concentration of 150 μg/mL.

### A549 Cell Culture

The A549 human lung carcinoma cell line was purchased from Sigma-Aldrich. Cells were cultured in high glucose Dulbecco’s Modified Eagle Medium (DMEM) supplemented with 10% fetal bovine serum (FBS) and 1% penicillin–streptomycin (all from Gibco) in a humidified incubator at 37 °C with 5% CO_2_. Cells were routinely subcultured to maintain an exponential growth.

A549 cells were seeded at 10,000 cells per well in a 96-well plate and incubated for 20 h for cell adhesion. After washing with PBS, cells were incubated with nanoparticles at concentrations ranging from 200 to 12.5 μg/mL in fresh media for 20 h to study their cytotoxicity.

### LDH Cytotoxicity Assay

Cell viability was determined through the evaluation of lactate dehydrogenase (LDH) activity using the Cytotoxicity Detection kit (Roche), in accordance with the manufacturer’s protocol. After incubation with nanoparticles, cell supernatant was collected for the measurement of LDH activity. 1% triton X-100 was used as positive/high control, causing 100% cell cytotoxicity, and untreated cells as negative/low control, with no cell cytotoxicity. The reaction was stopped after 15 min, and the absorbance was measured at 490 nm using a microplate reader.

### Alamar Blue Cell Viability Assay

After 20 h of incubation with nanoparticles, fresh medium containing Alamar Blue reagent (Thermofisher Scientific) was added to each well according to the assay protocol. The cells were further incubated for 4 h to allow the reduction of resazurin (alamarBlue) to the fluorescent product resorufin by metabolically active cells. Fluorescence intensity was measured using a microplate reader with excitation and emission wavelengths appropriate for Alamar Blue (e.g., excitation at 570 nm and emission at 600 nm). Untreated cells were used as positive control with 100% viability, and media with no cells was used as negative control with zero metabolic activity. Cell viability was calculated relative to control wells, and the results were expressed as a percentage of viable cells.

### Model Antigen Loading

Ovalbumin (OVA) supplied by invivogen was utilized as a model antigen for vaccine conjugate testing. To evaluate the loading capacity of calcium phosphate (CaP) nanoparticles, increasing concentrations (50 μg/mL, 100 μg/mL, 250 μg/mL, and 500 μg/mL) of OVA were combined with a final NPs concentration of 500 μg/mL. After 20 h of stirring using a roller mixer at room temperature, the samples were centrifuged to effectively separate the unconjugated OVA from the NP-OVA conjugate. Pierce BCA Protein Assay (Thermo Scientific) was used to quantify the OVA concentration as per the supplier’s protocol in the supernatant, allowing the estimation of OVA adsorbed on the surface of CaP NPs.

### Proteinase K Degradation Assay

For OVA stability of the OVA, the three different CaP nanoparticle-OVA conjugate samples were prepared with a final concentration of 200 μg/mL of OVA and 2 mg/mL of CaP NPs, i.e., a 1:10 ratio of the OVA to CaP upon overnight mixing on the roller mixer. Protein stability was tested by incubating 3 μg of OVA, either alone or conjugated with three different types of CaP NPs, with 20 ng of proteinase K (Qiagen) in 20 mM Tris–HCl pH 8.0. Samples were incubated at 37 °C for up to 6h. The enzymatic reaction was stopped at different time points by the addition of a lithium dodecyl sulfate (LDS) sample buffer supplemented with β-mercaptoethanol (Invitrogen). Samples were subjected to sodium dodecyl sulfate-polyacrylamide gel electrophoresis (SDS-PAGE) using 4–12% Bis–Tris gels (Invitrogen) and MES buffers (Invitrogen). Gels were washed with water and stained with Imperial Protein Stain (Thermo Scientific). Images were acquired on a GelDoc XRS+.

### Bone Marrow-Derived Dendritic Cell Culture

Murine bone marrow-derived dendritic cells (BMDCs) were generated by isolating bone marrow cells from the femurs and tibias of 6–8-week-old C57BL/6 male mice. Bone marrow from two mice was pooled for each biological replicate. 2 × 10^6^ cells were cultured in RPMI-1640 (Gibco) medium supplemented with 10% fetal bovine serum (FBS), l-glutamine (Gibco), 1% penicillin–streptomycin, and 20 ng/mL granulocyte-macrophage colony-stimulating factor (GM-CSF) from PeproTech. Media was refreshed on days 3, 6, and 8 before collecting loosely and nonadhered BMDC on day 10 for subsequent experiments.

### Nanovaccine Conjugate Testing

10^6^ BMDCs were seeded per well of a 24-well plate and were stimulated for 18 h with 10 μg of OVA conjugated with and without CaP NPs in the presence of 5 ng/mL GM-CSF. The CaP-OVA conjugate samples were prepared with the same composition as the OVA stability assessment with a 3 h mixing period. Supernatants were stored at −20 °C for subsequent cytokine analysis by ELISA. For the OVA uptake and processing assays, BMDC were stimulated for 20 min at 37 °C with CaP nanoparticles conjugated with OVA-Texas Red (Invitrogen) and DQ-OVA (Invitrogen) respectively.

### Flow Cytometry

After stimulation, BMDC were collected and washed in PBS before being stained with Zombie UV viability dye (Biolegend) for 15 min at room temperature to exclude dead cells from the analysis. To characterize BMDC activation, cells were incubated with antibodies against CD11c (N418, Biolegend), CD80 (16-10A1, Biolegend), CD86 (CLONE: GL-1, Biolegend), and CD40 (1C10, eBioscience Invitrogen) in the presence of an FcR block (Biolegend) for 25 min at 4 °C in the dark. OVA uptake and processing assays were analyzed using BD Fortessa, and 18 h stimulated cells were analyzed using SONY ID700. Data were analyzed using FlowJo software (Treestar), and fluorescence minus one (FMO) controls were used to set the gates.

### ELISA Measurements

Cytokines IL-4, IL-10, TNFα were quantified using Thermofisher ELISA kits, and cytokines IL-12p40 & IL-6 were quantified using BD biosciences ELISA kit as per manufacturer’s instructions.

### Ion Release Kinetics of Ca^2+^

Calcium ion release at 37 °C was evaluated over time by incubating nanoparticle suspensions (1 mg/mL in ultrapure water, sonicated for 5 min) for 0, 1, 2, 4, and 24 h. At each time point, samples were centrifuged to pellet the nanoparticles, and the supernatants were collected and stored at 4 °C. Calcium concentrations were measured using inductively coupled plasma optical emission spectroscopy (ICP-OES, Agilent 5110, radial mode) after acidifying 0.5 mL of each supernatant with 4.5 mL of 2% HNO_3_. External calibration was performed by using certified calcium standards (Inorganic Ventures).

### Statistical Analysis

All of the data are plotted as the mean ± standard error mean (SEM) of three independent biological experiments unless stated otherwise. One-way analysis of variance (ANOVA) was performed for comparison using GraphPad prism. A *p* value of <0.05 was considered for statistical significance (single star).

## Results and Discussion

### Flame-Made CaP Nanoparticle Morphology

Figure 1 provides a schematic overview of the nanoparticle synthesis and evaluation strategy, outlining the generation of three calcium phosphate nanoparticle (CaP NP) formulations with distinct sizes. Flame nanoparticle synthesis allows for a fine control over the average nanoparticle size by tuning the process conditions.^30,34,35^ More specifically, by controlling the particle residence time at high temperatures as well as the precursor concentration in the flame, the average primary particle size can be finely tuned.^36–38^

**Figure 1:**
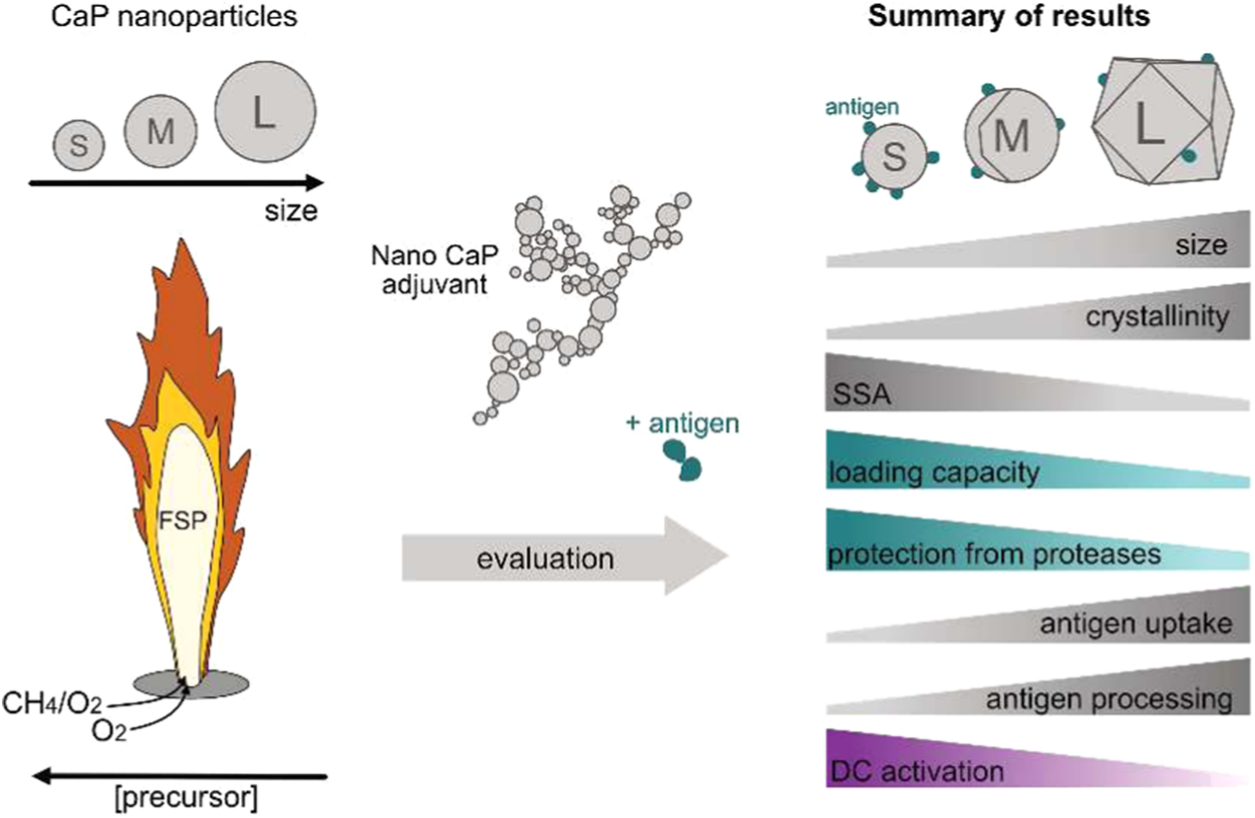
Schematic illustration of the flame spray pyrolysis (FSP) process used for the synthesis of calcium phosphate nanoparticles with three distinct sizes, achieved by tuning the precursor concentration and flame parameters. The illustration also shows key size-dependent trends observed across the formulations, summarizing their impact on the performance and biological outcomes.

This size-controllability is shown in Figure 2, in which three transmission electron microscopy (TEM) images are shown for three different process conditions (please see the Supporting Information, SI, and Table S1 for detailed conditions), yielding CaP nanoparticles with three distinct sizes, (A) small (S), (B) medium (M), and (C) large (L). In all three TEM images, the fractal-like nanoaggregate structure, characteristic for flame-made nanoparticles,^31,34,39^ was observed, with an average primary particle sizes of 14.4 nm for medium CaP nanoparticles and 19.5 nm for large CaP nanoparticles (for the small particles, it was not possible to measure the average size due to the structure) please see the SI, Figure S1, for particle size distributions. The size control from the different process conditions was further validated by the specific surface area (SSA) values as determined by N_2_ adsorption of all three samples as shown in Figure 2D, in which the SSA is plotted as a function of the precursor concentration in the flame (please note the average *d*_BET_ sizes for each sample in the plot, assuming monodisperse spherical particles of homogeneous density). The SSA of M CaP and S CaP is almost two and three times the one of L CaP, respectively. There is an inverse relationship between the specific surface area and precursor concentration in flame, consistent with the literature demonstrating a similar effect for flame-synthesized CeO_2_ NPs.^40^ Thus, by tuning the flame process conditions, fine control of the average primary particle size as well as the SSA of the samples can be achieved.

**Figure 2:**
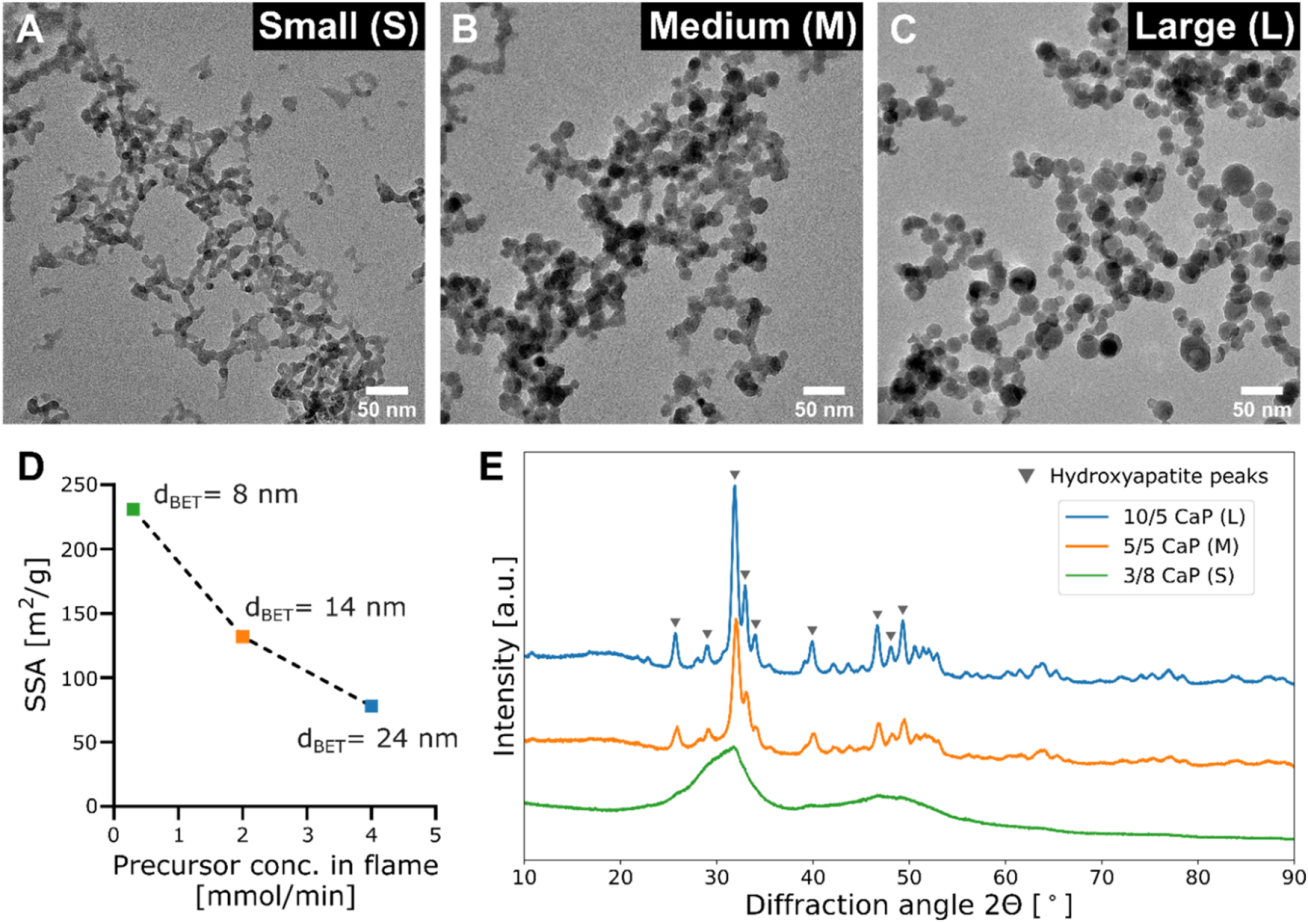
Three different calcium phosphate (CaP) nanoparticles (NPs), varying in particle size and crystallinity, were synthesized by using flame spray pyrolysis (FSP). TEM images showcasing the particle morphology and primary particle size of CaP NPs produced using three different process conditions: 3/8 flame, 5/5 flame, and 10/5 flame, generating (A) Small (S), (B) Medium (M), and (C) Large (L) sized nanoparticles, respectively. (D) Specific surface area of the as-synthesized NPs measured using the N_2_ gas adsorption isotherm as a function of precursor concentration in the flame during the particle synthesis as well as their primary particle size (*d*_BET_) estimated using Brunauer–Emmett–Teller (BET) analysis. (E) XRD patterns of the nanoparticles with the hydroxyapatite peak positions indicate the crystalline hydroxyapatite formation in the case of large and medium CaP NPs and broader peak denoting the highly amorphous nature of small CaP NPs.

In addition to the average primary particle size obtained for the three samples, the crystallinity of the as-produced CaP nanoparticles is also tunable. Figure 2E shows the X-ray diffraction (XRD) patterns of all three samples. The large (L) CaP nanoparticles (blue line) exhibited high crystallinity, with the resulting peak positions matching the pattern of hydroxyapatite (shown as inverted triangles, ICSD: 151414). The medium-sized (M) CaP nanoparticles (orange line) were also crystalline with characteristic hydroxyapatite peaks, albeit less than the large ones. Finally, the small (S) CaP nanoparticles (green line) exhibited only broad bands in their XRD pattern, indicating that this sample is amorphous. The nominal Ca/P ratio in all samples is identical at 2.19. The X-ray diffraction patterns, specific surface area (SSA), and primary particle size measurements of S CaP align with those reported by Tsikourkitoudi et al.,^33^ who employed a similar synthesis protocol. Similarly, the characteristics of L CaP correspond to findings by Merkl et al. using the same flame synthesis method,^32^ highlighting the reproducibility of CaP nanoparticle synthesis by FSP across batches. Furthermore, Ansari et al. recently demonstrated the scalability and batch-to-batch consistency of flame-synthesized nanoparticles at the pilot scale.^41^

### *In Vitro* Cytotoxicity and Biocompatibility

The biocompatibility of CaP nanoparticles is well-documented;^42^ here, we reassessed it by evaluating the cytotoxicity of the three CaP formulations against human lung epithelial cells (A549 cell line). The cells were incubated with increasing concentrations of nanoparticles for 20 h, and the CaP nanoparticle suspensions were sonicated prior to their incubation with the cells to ensure dispersibility. The hydrodynamic sizes of these nanoparticles in pure water were in the μm size range (please see the SI, Table S1), indicating that CaP nanoparticles agglomerate when dispersed in aqueous solutions in agreement with the literature.^33,40^ Figure 3A shows the cell viability for all three CaP nanoparticles with increasing concentration, as determined by the lactate dehydrogenase (LDH) assay, which monitors the membrane integrity. None of the CaP nanoparticles, be it amorphous or crystalline, induced cell membrane damage to A549 cells, even at the highest tested concentration of 200 μg/mL.

**Figure 3:**
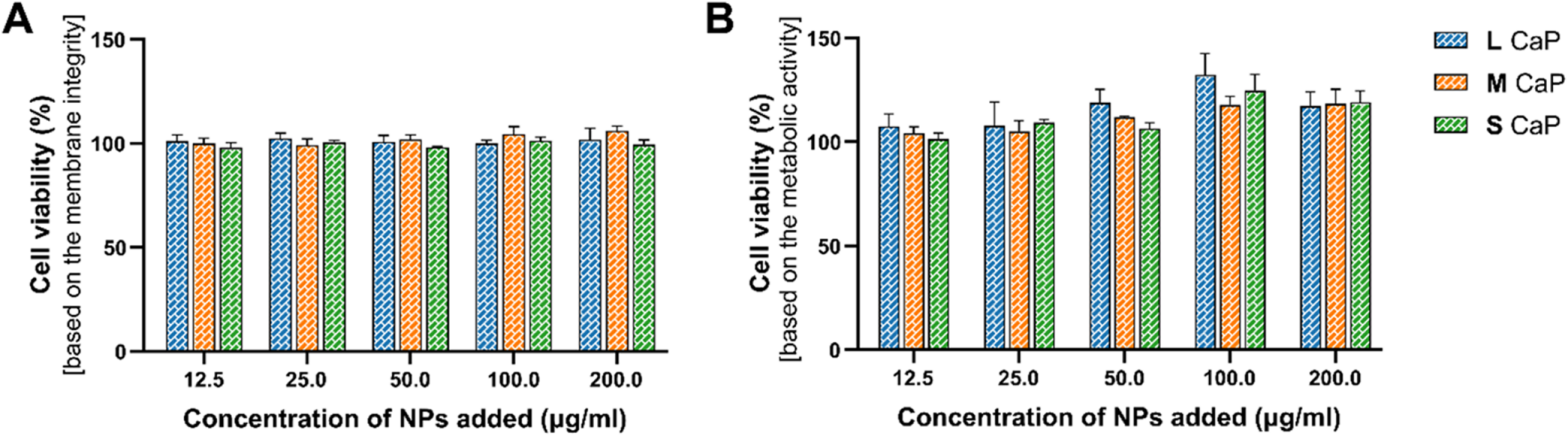
Human lung epithelial cell line (A549) was used for the assessment of dose-dependent (12.5 to 200 μg/mL) cytotoxicity of the CaP NPs over a 20 h exposure. *In vitro* cell viability of A549 evaluated using (A) the lactate dehydrogenase (LDH) activity of the NP-treated cells identifying membrane damage. 2% triton X-100 was used as negative (100% cytotoxicity) control (B) alamarBlue assay where the metabolic activity of the cells was estimated based on the reduction of resazurin based alamarBlue solution to resorufin (a fluorescent compound). Data are presented as the mean ± SEM from three independent biological experiments.

The biocompatibility of the as-prepared CaP nanoparticles was also examined by monitoring the metabolic activity of the cells measured by using the alamarBlue assay (Figure 3B). Consistent with the membrane integrity results, the as-prepared CaP nanoparticles did not affect the metabolic activity of the cells for all concentrations tested, indicating that the CaP nanoparticles synthesized by FSP are biocompatible. These findings align with the literature, where flame-synthesized amorphous CaP nanoparticles with varying Ca-to-P ratios showed no toxicity toward urine-derived stem cells at concentrations up to 50 μg/mL for up to 7 days *in vitro*.^31^ Rod-shaped hydroxyapatite have also been shown to have no *in vitro* cell cytotoxicity against bone marrow-dendritic cells (BMDCs) for concentrations 5 μg/mL and 10 μg/mL.^43^ Our findings further confirm that CaP is a highly biocompatible biomaterial and suitable to be investigated as a drug nanocarrier and vaccine adjuvant.^6,16,44^

### Biomolecule Loading and Stability against Enzymatic Degradation

Biomolecule antigen loading is one of the most important characteristics of adjuvants and nanocarriers. Figure 4A shows the loading capacity (mass of antigen per mass of CaP) of all three differently sized CaP nanoparticles with a model antigen, ovalbumin (OVA), as a function of the antigen concentration. OVA is widely studied as a model antigen in vaccine adjuvant studies due to the potent immunogenicity that is generated in its presence.^45^ All three nanoparticles demonstrated comparable loading capacities at low OVA concentrations. At the lowest OVA concentration of 50 μg/mL, the loading efficiency approaches 100% for each nanoparticle (please see the SI, Figure S2) with loading capacity of 100 μg of OVA per mg of CaP nanoparticles. However, as the OVA concentration increases, there is a clear size-dependent effect, with the S CaP nanoparticles exhibiting significantly higher loading capacities than the M CaP and L CaP nanoparticles (please see the SI, Figure S3 for dissolution data). This could be attributed to the higher SSA of the small nanoparticles compared to the medium and large ones. For the S CaP nanoparticles, the loading capacity reached ∼500 μg/mg of NPs, demonstrating the high drug-loading potential of flame-made nanocarriers. In contrast, M CaP and L CaP nanoparticles showed a plateau in loading capacity at higher OVA concentrations likely due to surface saturation.

**Figure 4:**
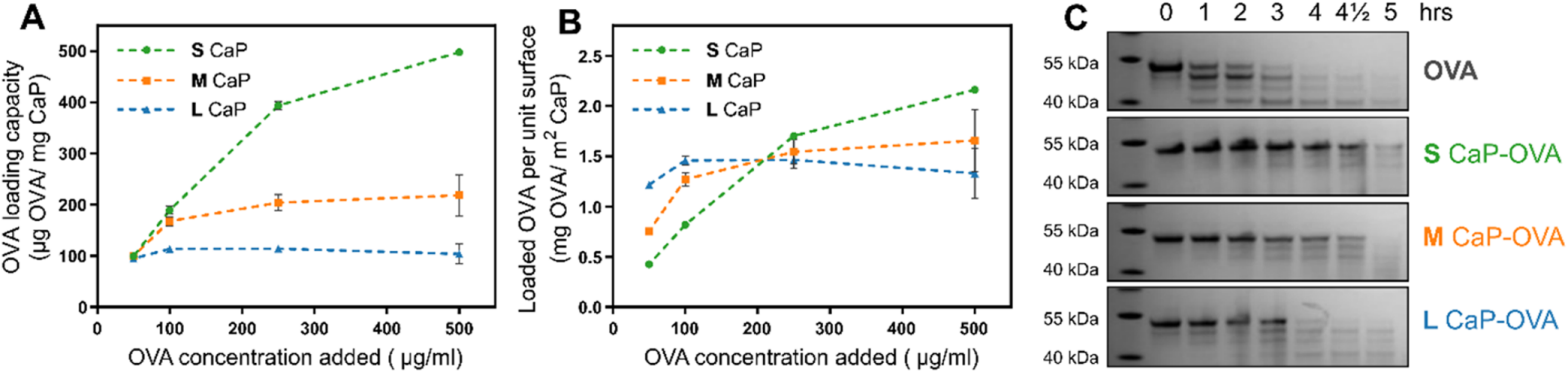
Ovalbumin (OVA), serving as a model antigen, was loaded on the surface of CaP NPs by physisorption, and the stability of OVA conjugated with CaP NPs was assessed with proteinase K degradation assay. (A) OVA loading capacity of different CaP nanoparticles and (B) OVA adsorption per unit surface area after overnight incubation with increasing (50 to 500 μg/mL) concentrations of ovalbumin. Data are presented as the mean ± SEM, *n* = 3. (C) SDS-PAGE gel showing the proteolytic degradation of free/pure OVA compared with that of OVA loaded on different NPs at different incubation times with proteinase K.

This plateau is further confirmed in Figure 4B, which shows the amount of OVA loaded per surface area. M CaP and L CaP reached a saturation point at ∼1.5 mg of OVA/m^2^, indicating that antigen loading is directly proportional to available surface area. However, S CaP continued to exhibit increasing OVA loading, suggesting additional contributing factors, such as its amorphous nature and reduced steric hindrance. The smaller primary particle size of S CaP likely minimizes steric constraints, improving surface accessibility for OVA adsorption, further explaining the absence of a clear saturation point up to the tested OVA concentrations. Table 1 shows the comparison of S CaP loading efficiency and loading capacity with the drug carriers in the literature, confirming that S CaP outperforms most other nanocarriers reported in the literature in terms of both loading capacity and loading efficiency. Only mesoporous silica nanoparticles could demonstrate a higher loading capacity for OVA of around 600 μg/mg.

**Table 1:** Comparison of Different Nanoparticle Systems from the Literature Based on their OVA Loading Performance and BMDC Activation on Their Own or Along with Additional Immunostimulant^a^

		ovalbumin loading		immunological response (BMDC)		
nanoparticle system	additional immunostimulant	loading capacity (μg OVA/mg of NP)	loading efficiency (%)	fold change in MFI	% increment in activated DCs	references
silica solid sphere	CpG	458 μg/mg			CD86^+^ = 40%	^50^
					CD80^+^ = 40%	
					CD40^+^ = 80%	
mesoporous silica (MSNs-L)		604 μg/mg	89.6%	CD86 < 1.5 fold		^56^
				CD80 < 1.5 fold		
CaCO_3_	pneumolysin	60 μg/mg	90%		CD86^+^ = 28%	^45^
					CD80^+^ = 45%	
					CD40^+^ = 16%	
hybrid nanovesicles (protein antigen-lipid nanovesicles)	MPL-A	180 μg/mg (18% loading yield)	93%	CD86 = ∼3.5 fold	CD86^+^ = ∼10%	^57^
				CD80 = ∼2.3 fold	CD80^+^ < 2%	
				CD40 = ∼2.4 fold	CD40^+^= ∼14%	
PLGA		34.8 μg/mg		CD86 = ∼2 fold		^58^
				CD80 < 1.5 fold		
ZIF-8 NPs (Zn^2+^:2-MIM)		13.1 μg/mg (1.3% loading content)	97%	CD86 = ∼2.7 fold	CD86^+^ = ∼36%	^59^
				CD40 = ∼3 fold	CD40^+^ = 31%	
hollow ZIF-8 (HZIF-Mn)	Mn^2+^		75%		CD86^+^ = 20%	^60^
					CD80^+^ = ∼39%	
lipid-coated mesoporous silica	MPL-A		78%	CD40 < 1.3 fold		^61^
metal-phenolic networks	CpG	∼210 μg/mg	49.6%		combined CD80^+^CD86^+^ = ∼20%	^62^
calcium phosphate (S CaP)		500 μg/mg	100%	CD86 = 17 fold	CD86^+^ = 55%	this work
				CD80 = 2.5 fold	CD80^+^ = 41%	
				CD40 = 9 fold	CD40^+^= 26%	

a OVA loading is reported as either loading capacity (μg OVA/mg of NP) or loading efficiency (%). Dendritic cell activation is reported as the fold change in MFI of the reported maturation markers (CD86, CD80, and CD40) and percentage increase in the DC population expressing each of these maturation markers compared to controls. The best nanoparticle systems were chosen in case of multiple formulations reported in these research articles.

Another important characteristic of nanocarriers is their ability to extend antigen bioavailability by protecting the loaded antigen from enzymatic degradation.^46^ We investigated this by incubating pure OVA and OVA loaded onto all three CaP nanoparticle samples, each with an identical amount of OVA (3 μg), in the presence of proteinase K, an enzyme known for rapid protein degradation. Subsequently, we assessed OVA stability at different incubation times using SDS-PAGE gel, as shown in Figure 4C. Initially, at 0 h prior to enzyme addition, all samples exhibited a single, strong band corresponding to OVA’s molecular weight (43 kDa), indicating that OVA is intact for all samples. However, pure OVA began to degrade after just 1 h incubation in the presence of proteinase K, with the OVA band disappearing completely after 3 h. In contrast, when the OVA was loaded onto the CaP nanoparticles, the OVA band persisted for longer incubation periods, resisting degradation for at least 3 h with all nanoparticles. This highlights the ability of these CaP nanoparticles to protect large biomolecules from enzymatic degradation. This observation aligns with existing literature, in which it has been shown that flame-made CaP nanoparticles protect peptides from enzymatic degradation;^33^ however, this is the first time that a similar protection is validated for larger biomolecules, such as protein antigens. Interestingly, the S CaP nanoparticles offer the longest protection of the OVA against the enzymatic degradation, with the M and L CaP nanoparticles providing decreasing protection.

### Cellular Uptake and Processing of Ovalbumin

Immunomodulatory effects of CaP nanoparticles-OVA conjugates were studied using CD11c expressing live murine bone marrow-derived dendritic cells (BMDCs) (please see the SI for the flow cytometry event gating, Figures S4 and S5). Dendritic cells (DCs) play a crucial role in innate immunity as primary antigen-presenting cells. They are essential for initiating and modulating adaptive immune responses by efficiently internalizing antigens, processing them intracellularly, and subsequently presenting them to T cells.^47–49^ To assess antigen uptake by DCs, we employed an OVA conjugated with Texas Red fluorescent dye (OVA_TR_). Figure 5A illustrates the percentage of total CD11c^+^ DCs that have internalized fluorescent OVA. While over 80% of DCs could uptake free OVA, a noticeable increasing trend in the percentage of OVA_TR_^+^ DCs can be observed for CaP nanoparticles-OVA samples, particularly with L CaP nanoparticles exhibiting maximum OVA_TR_^+^ DCs. Figure 5B highlights the increase in the overall level of uptake of the OVA by DCs for CaP-OVA samples, as indicated by the fold change in total mean fluorescence intensity (MFI) compared to that of the OVA alone. OVA with CaP leads to higher antigen uptake per DC, as the percentage of DCs internalizing OVA_TR_ remains similar. All three nanoparticles enhanced the OVA internalization, specifically L and M CaP nanoparticles significantly augmented the uptake by more than twofold. This proves that the conjugation of OVA to the CaP nanoparticles enhanced its delivery to the DCs, which was also seen previously for other nanoparticle systems.^45,50^

**Figure 5:**
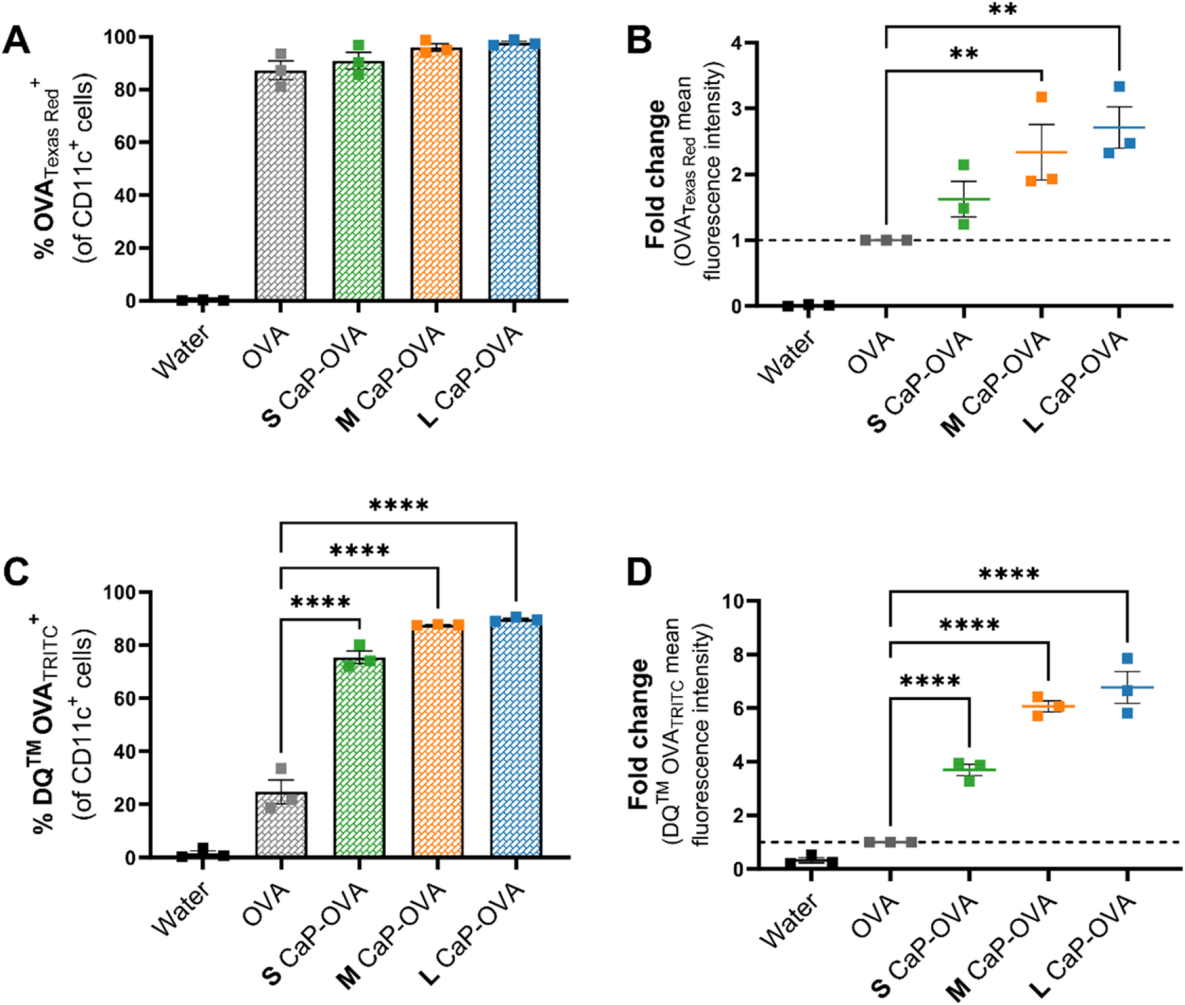
OVA uptake and processing by CD11c^+^ cells (DCs) were quantified by flow cytometry. Texas Red and BODIPY (DQ)-labeled OVA conjugated with CaP NPs were incubated with the DCs for 20 min at 37 °C, 5% CO2, and 95% humidity. (A) Percentage of DCs taking up the Texas Red-labeled OVA (OVA_TR_) conjugated with CaP NPs compared to free OVA_TR_ (B) Fold change in the mean fluorescence intensity from OVA_TR_ taken up by the DCs when delivered with and without the CaP NPs. DQ-OVA exhibits TRITC fluorescence upon accumulation in an acidic environment (C) Percentage of DCs exhibiting fluorescence linked with DQ-OVA, indicating the antigen processing, and (D) fold change in the mean fluorescence intensity from DQ-OVA when delivered with and without different CaP NPs. Data are presented as the mean ± SEM from three independent biological experiments. One-way ANOVA was performed with all samples compared to OVA, *p < 0.05, **p < 0.01, ***p < 0.001, ****p < 0.0001.

Following the cellular uptake, the antigen must be processed within the endo/lysosomal compartment to enable successful presentation by the DCs via MHC II pathway.^51^ Therefore, we evaluated antigen processing by DCs using OVA labeled with BODIPY dye (DQ-OVA), a pH insensitive, self-quenched conjugate that fluoresces upon proteolysis.^52,53^ Figure 5C shows the percentage of CD11c^+^ DCs displaying the fluorescence signals attributed to DQ-OVA. All three CaP nanoparticle samples greatly enhance the processing of the OVA by DCs, with approximately 50% more cells exhibiting fluorescence. Figure 5D illustrates the fold change in DQ-OVA fluorescence, revealing an almost fourfold increase in antigen processing with S CaP nanoparticles compared with that in OVA alone. The conjugation of OVA with CaP nanoparticles not only increased the percentage of DCs processing the antigen but also significantly boosted the total amount of antigen processing. Furthermore, M and L CaP nanoparticles exhibited an even higher enhancement, with the increase exceeding sixfold. The higher increase in case the M and L CaP nanoparticles compared to S CaP particles can be attributed to the significant increase in the OVA uptake when conjugated with M CaP and L CaP in the first 20 min, which could further aid in the OVA processing by BMDCs in case of these nanoparticles. Another possible explanation is that the ability of S CaP nanoparticles to resist OVA degradation for longer periods as shown with the proteinase K assay (Figure 4C) which could lead to slower antigen processing in the first 20 min, resulting in lower DQ-OVA MFI fold change compared to M and L CaP. These results indicate that CaP nanoparticles not only enhance the antigen internalization by DCs but also their subsequent processing, which is crucial for their presentation to CD4^+^ T cells via MHC II. This highlights the potential of flame-synthesized CaP nanoparticles as a powerful tool in antigen delivery.

### Dendritic Cells Activation and Stimulation

Recombinant proteins as antigens are generally poorly immunogenic, which is why they need immunopotentiators to induce a protective immune response.^1,3^ Therefore, we investigated the immunostimulatory properties of the CaP nanoparticles by studying the activation and maturation of DCs through the upregulation of the costimulatory markers CD86, CD80, and CD40, which are important for downstream activation of T cells.^47,48^ Figure 6 shows the enhancement in the percentage of CD11c^+^ DCs expressing these markers: (A) CD86, (B) CD80, and (C) CD40, after 18 h of incubation with free OVA, CaP nanoparticles alone or conjugated with OVA (please see the SI, Figure S6 for flow cytometry gating). Free OVA induces a basal level of DC activation comparable to the negative control (water), highlighting the inherently low immunogenicity of recombinant protein antigens. However, when the same antigen (OVA) was delivered in conjugation with CaP nanoparticles (CaP-OVA conjugates), there was a notable increase in the activation of DCs. Both the percentage of CD86 expressing DCs (shown in Figure 6A) and overall CD86 expression (shown in the SI, Figure S7) were upregulated for all three CaP-OVA conjugates. A significant enhancement was observed for S CaP-OVA conjugates compared to both free OVA and bare S CaP nanoparticles with a 16-fold increase in CD86 MFI (SI, Figure S7). However, the bare crystalline nanoparticles, M CaP and L CaP, showed similar enhancement as their OVA-conjugated counterparts. The same trend was observed in the case of CD80 marker, where all the CaP-OVA conjugates increased the CD80 expression, with the significant activation again obtained for the OVA-conjugated S CaP nanoparticles. Similarly, S CaP nanoparticles without OVA did not stimulate the CD80 expression by DCs. This indicates antigen-dependent immunostimulatory properties of S CaP nanoparticles, which are amorphous in nature. On the contrary, the M CaP and L CaP nanoparticles which are crystalline (hydroxyapatite) demonstrate the adjuvant properties independent of antigen presence. The slight immunogenic nature of hydroxyapatite rods has been reported in the literature independent of antigen as they stimulated the cytokine secretion by BMDCs.^43^ Regarding CD40 expression, although there is only a slight increase in the percentage of CD40^+^ dendritic cells with all of the CaP-OVA conjugates, the S CaP-OVA conjugate exhibits an eightfold increase in CD40 expression (SI, Figure S7). Table 1 shows that S CaP-OVA performs considerably better in DC activation without any additional immunostimulant compared to various nanoparticle systems reported in the literature.

**Figure 6:**
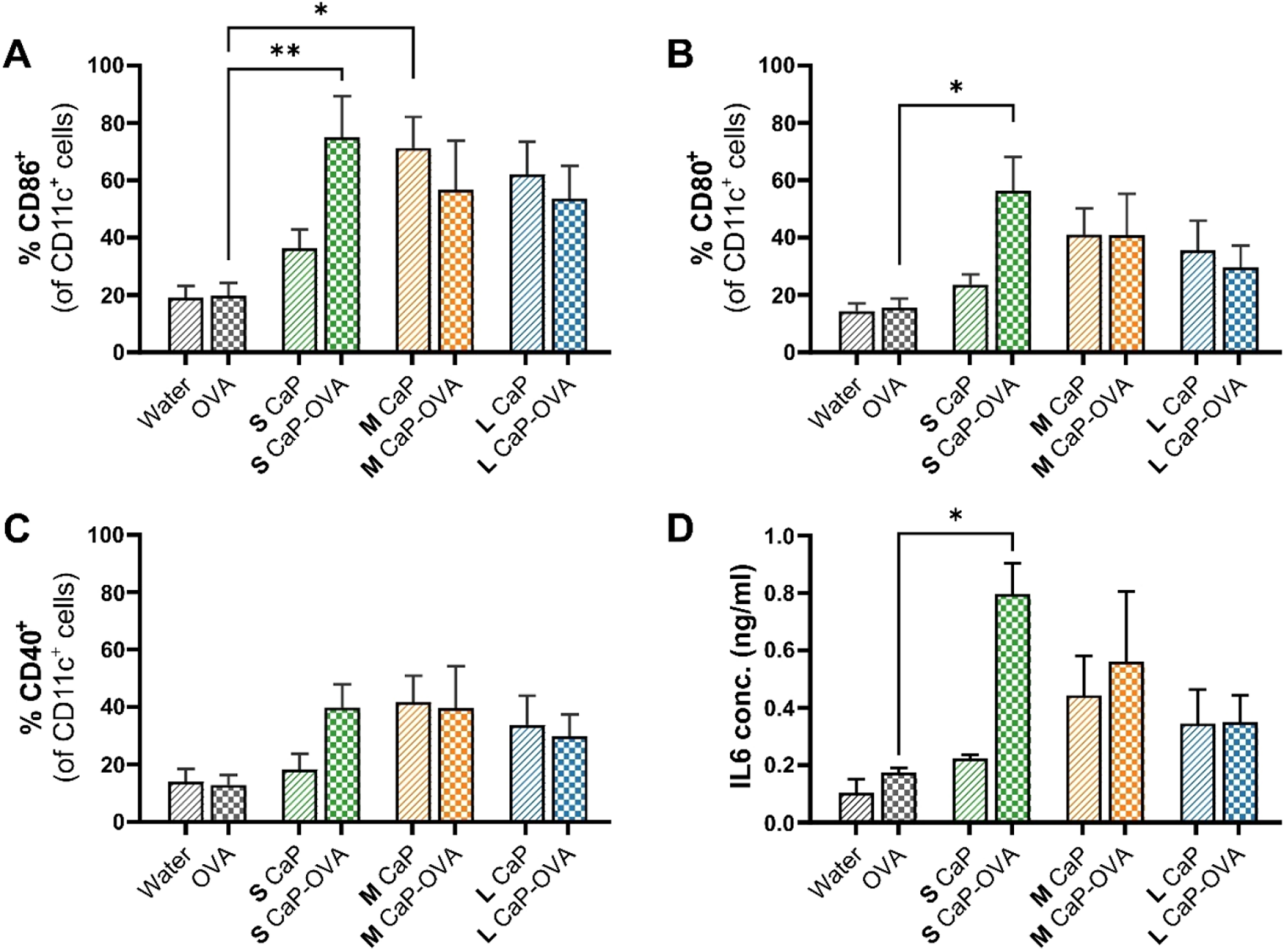
Phenotypic activation of DCs was assessed by flow cytometry and cytokine production by ELISA. Comparison of percentage of DCs expressing the (A) CD86, (B) CD80, and (C) CD40 surface markers upon 18 h stimulation with different CaP NPs with and without OVA. Bars with check pattern represent the data for samples conjugated with OVA and the bars with slant lines represent the data for samples without OVA. Control samples contain the cells stimulated with water or OVA. (D) IL-6 cytokine quantification from the supernatant of the stimulated DCs. Data are presented as the mean+SEM from four independent biological experiments (except for IL-6 with three independent biological experiments). One-way ANOVA was performed with all samples compared to OVA. *p < 0.05, **p < 0.01.

Apart from the upregulation of costimulatory markers, mature DCs also secrete cytokines which direct the appropriate T cell activation and polarization.^1^ IL-6 is a proinflammatory cytokine which is involved in the induction of a Th17 response, important for extracellular bacteria and fungi clearance.^54,55^ OVA-conjugated S CaP significantly increases IL-6 production compared to that of OVA or S CaP alone as shown in Figure 6D. In contrast, crystalline nanoparticles M CaP and L CaP, along with their antigen-conjugated formulations, exhibited a nonsignificant increase in IL-6. Additionally, these crystalline nanoparticles showed a slightly higher increase in secretion of proinflammatory cytokines, IL-12p40 and TNF-α, compared to amorphous S CaP (shown in the SI, Figure S8); however, the increase was not significant compared to that of OVA alone. Furthermore, none of the three nanoparticles were able to induce the production of the Th2-inducing cytokine, IL-4, or the immune regulatory cytokine, IL-10.^50,55^

Overall, these results indicate that all three CaP nanoparticles exhibit attractive properties as adjuvants, with the small (*d*_BET_ = 8 nm), amorphous CaP nanoparticles outperforming the M and L CaP that are crystalline when conjugated with OVA. On the other hand, the crystalline CaP nanoparticles enhanced the DC activation independent of antigen. While these findings highlight the influence of crystallinity on immunostimulatory effects, further *in vivo* studies are essential to evaluate the resulting antibody production and T cell responses and comparing with traditional adjuvants (e.g., alum-based), thereby confirming the potential of these nanoparticles as effective vaccine adjuvants.

## Conclusions

In this study, we address a research gap in the development of simple and scalable vaccine adjuvants by investigating the potential of flame-made calcium phosphate (CaP) nanoparticles. Our research demonstrates that flame spray pyrolysis (FSP) can produce biocompatible CaP nanoparticles with tunable sizes and crystallinity. We demonstrate that small amorphous CaP nanoparticles offer higher antigen loading capacities and enhanced protection against enzymatic degradation compared to their larger crystalline counterparts. Further, we demonstrated that the crystallinity of CaP nanoparticles plays a role in dictating their immunostimulatory properties. The crystalline nanoparticles M and L CaP showed better performance in the enhancement of antigen internalization and processing by dendritic cells compared to amorphous CaP nanoparticles. On the other hand, amorphous CaP nanoparticles proved to be superior for dendritic cell activation demonstrated by the antigen-dependent upregulation of CD86, CD80, and CD40 costimulatory receptors and IL-6 cytokine. These results highlight the potential of CaP nanoparticles as vaccine adjuvants, functioning not only as delivery agents but also as immunopotentiators. Additionally, the scalability and reproducibility of our flame synthesis method offer a significant advantage, enabling cost-effective and large-scale production, which is essential for meeting the demands of modern vaccine manufacturing. This study lays the foundation for the further development of flame-made CaP nanoparticles as adjuvants with immunostimulatory properties, prompting further research to establish their effectiveness and safety *in vivo*.

## Supplementary Material



## Notes

Detailed nanoparticle synthesis conditions along with their characterization using BET, DLS, and ELS; TEM primary particle size distribution; percentage of OVA loading efficiency; dissolution kinetics of NPs; Flow cytometry plots showing the gating strategy for OVA uptake, processing, and DC activation; fold change in upregulation of CD86, CD80, and CD40 markers during DC activation; and IL-12p40 and TNF- α cytokine quantification (PDF)
