# Supplementary material for: In Vitro Evaluation of Flame-Made Calcium Phosphate Nanoparticles for Antigen Delivery and Immunostimulation
Source: ACS Appl Nano Mater. 2025 May 27;8(23):11986–96. doi: 10.1021/acsanm.5c01535 (PMC12172012; doi:10.1021/acsanm.5c01535)
Supplement: Supplementary file 1 [file an5c01535_si_001.pdf]

SUPPORTING INFORMATION

In Vitro Evaluation of Flame-Made Calcium Phosphate Nanoparticles for Antigen Delivery and Immunostimulation

Anshika Maheshwari[a], Rebecca Dookie[a], Meztlli O. Gaytán[a], Birgitta Henriques-Normark[a,b] and Georgios A. Sotiriou[a,c*]

[a] Department of Microbiology, Tumor and Cell Biology, Karolinska Institutet, Stockholm, 171 77, Sweden.

[b] Clinical Microbiology, Karolinska University Hospital, Stockholm, 171 77, Sweden.

[c] Department of Chemistry, Science for Life Laboratory, Stockholm University, Stockholm 114 18, Sweden.

[*]Correspondence to: georgios.sotiriou@su.se

*Table **S1**: NP synthesis conditions (flame condition and precursor molarity) using flame spray pyrolysis for three different sizes of Calcium phosphate (CaP) nanoparticles along with their specific surface area, primary particle size estimated using Brunauer–Emmett–Teller (BET) analysis, mean hydrodynamic size and zeta potential measure using dynamic light scattering.*

**Flame condition (p/d)** Precursor flow (mL/min) **/** dispersion oxygen (L/min)	**Precursor molarity** (M)	**Precursor concentration in flame** (mmol/min)	**SSA** (m^2/g)	**d_{BET}** (nm)	**Mean hydrodynamic size** (nm)	**ζ potential** (mV)
3/8 CaP (**S**)	0.1	0.3	231	8	2571	- 3.5
5/5 CaP (**M**)	0.4	2.0	132	14	1406	- 7
10/5 CaP (**L**)	0.4	4.0	78	24	1209	- 3.9

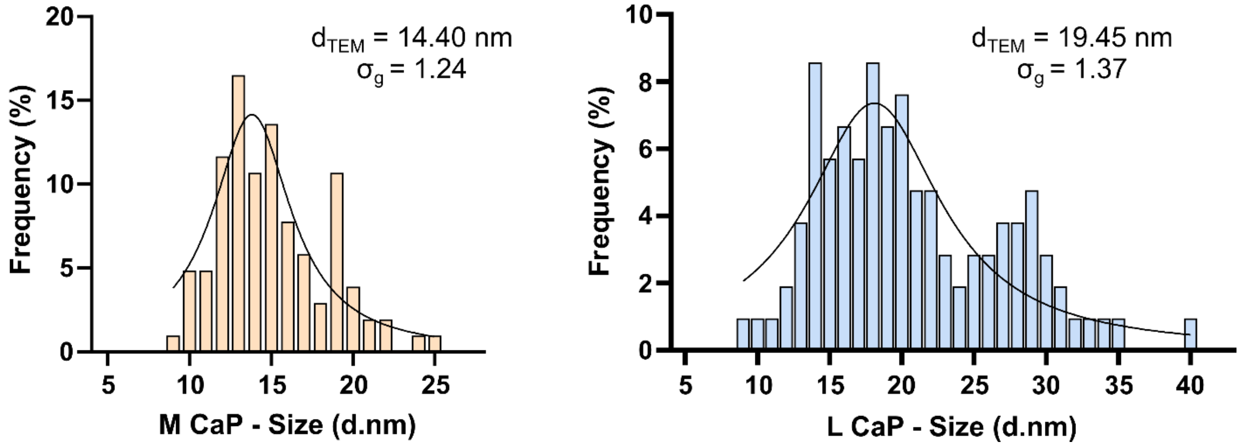

*Figure **S1**: Primary particle size distribution of M and L CaP as measured from the TEM images of each NPs. Mean diameter (d_{TEM}) and geometric standard deviation (σ_g) of the particles as per the log-normal fitting are mentioned of each NP.*

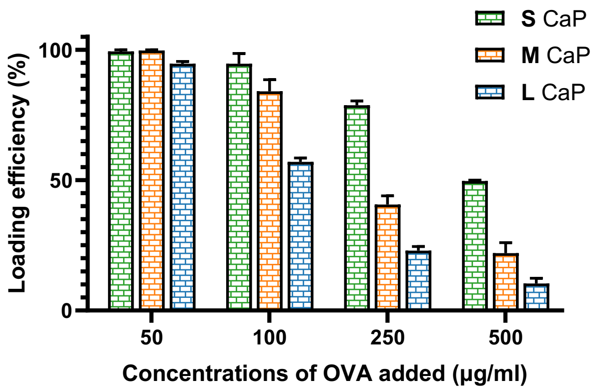

*Figure **S2**: Loading efficiency of S, M and L CaP nanoparticles upon overnight incubation with 4 different concentrations of ovalbumin.*

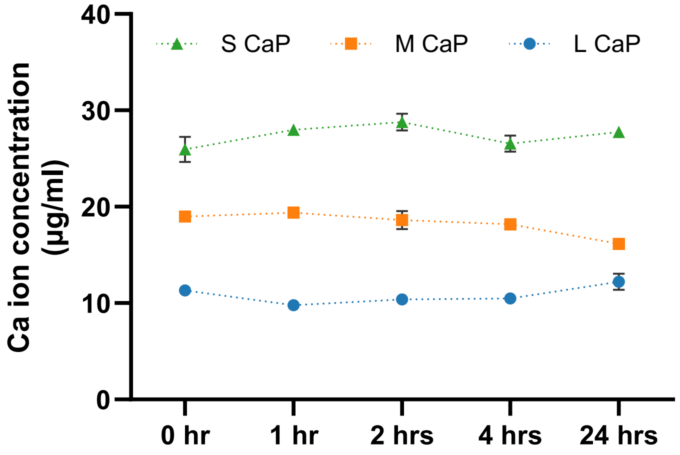

*Figure **S3**: Calcium ion release profile from small (S), medium (M) and large (L) CaP nanoparticles at 37°C with time. Ion concentrations in the supernatant were quantified by inductively coupled plasma optical emission spectroscopy (ICP-OES).*

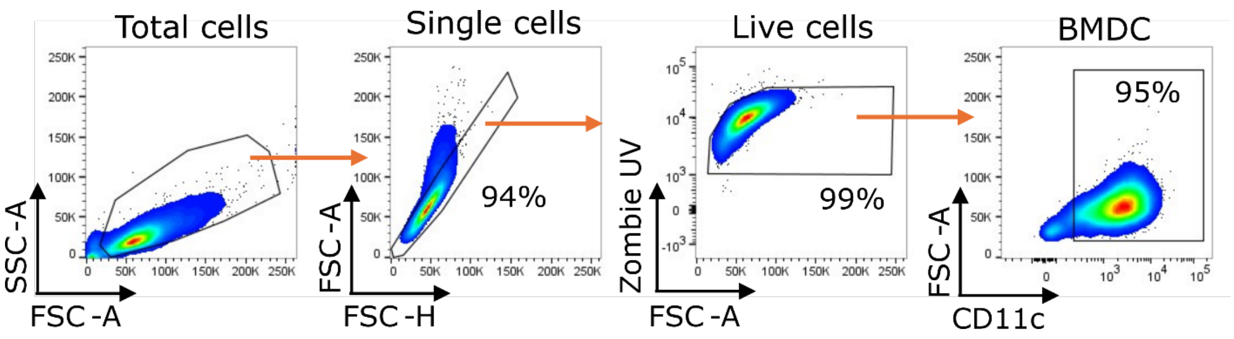

*Figure **S4**: Flow cytometry panels showing the gating of BMDC to select the final CD11c+ population for antigen uptake and DC activation analysis.*

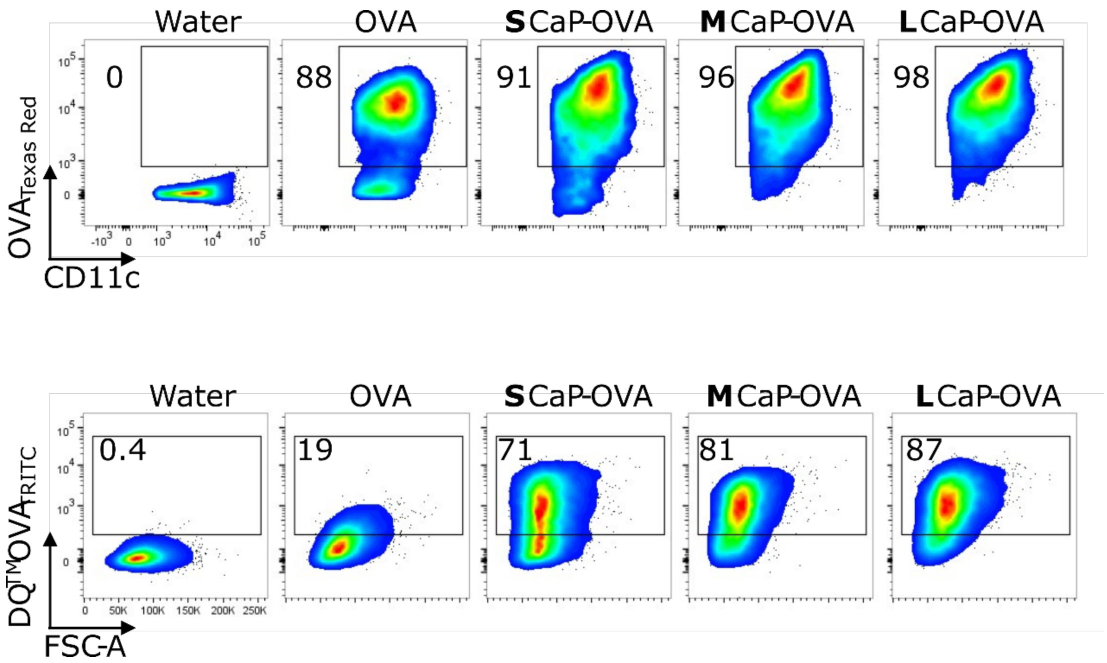

*Figure **S5**: Representative flow cytometry plots showing gating and the percentage of DCs exhibiting fluorescence associated with Texas Red and TRITC DQ-OVA, indicating antigen uptake and processing, respectively, for S, M, and L CaP nanoparticles.*

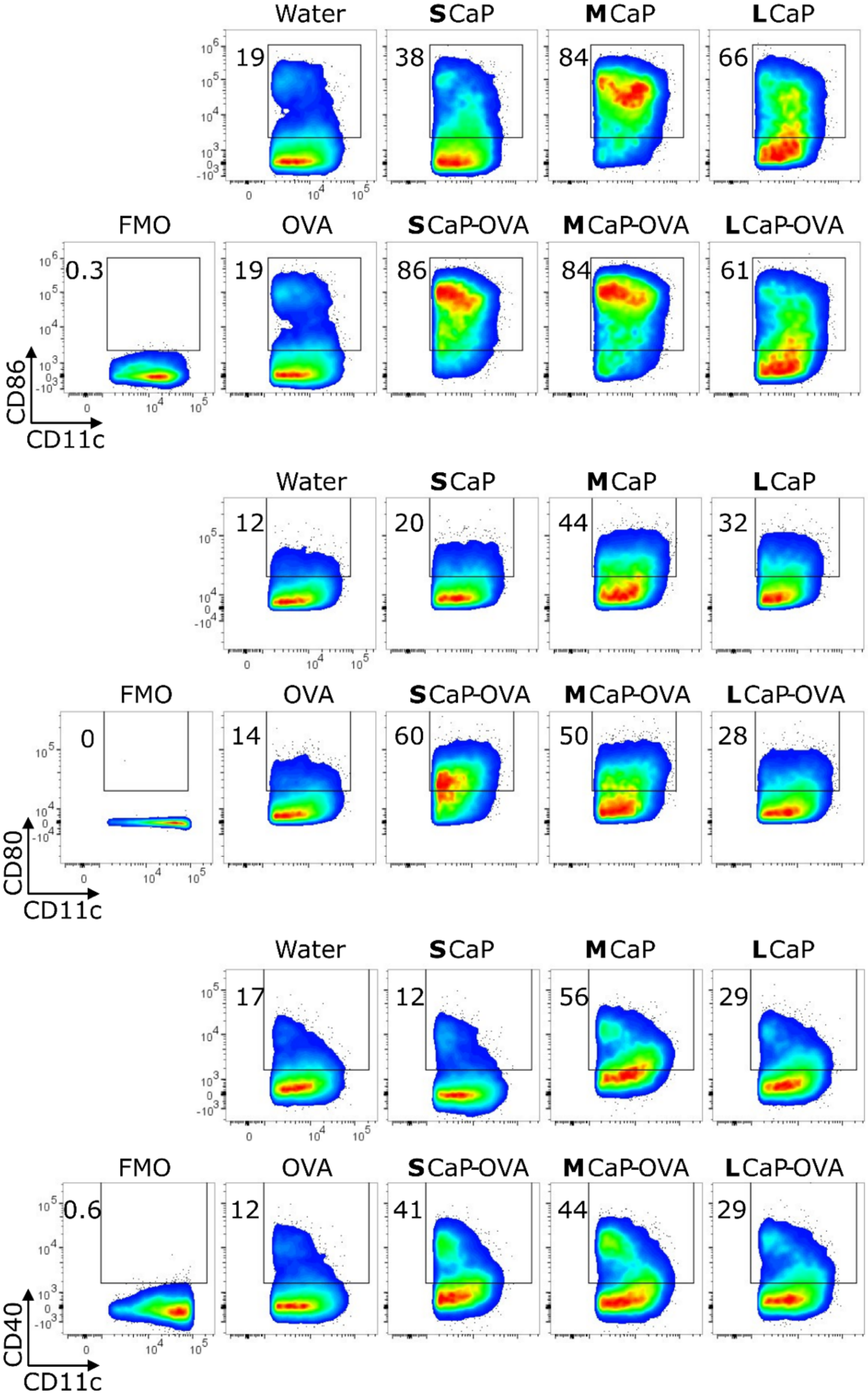

*Figure **S6**: Flow cytometry plots from representative experiment showing gating strategy using FMO control for the percentage of BMDCs expressing CD86, CD80 and CD40, after 18 hrs stimulation with different CaP NPs with and without OVA.*

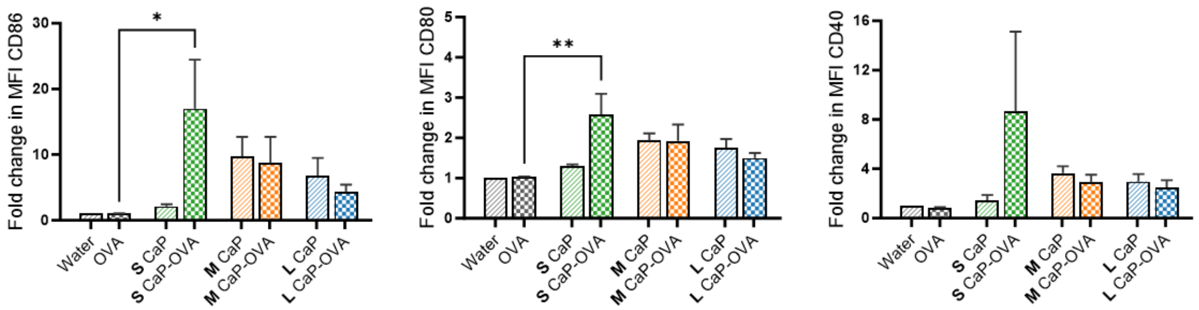

*Figure **S7**: Fold change in the upregulation of co-stimulatory markers CD86, CD80 and CD40 on BMDCs upon activation after 18 hrs stimulation with different CaP NPs with and without OVA. Bars with check pattern represent the data for samples conjugated with OVA and the bars with slant lines represent the data for samples without OVA. Control samples contain the cells stimulated with water or OVA. Fold change calculated with respect to the water samples. Data is presented as the mean+SEM from 4 independent biological experiments. One-way ANOVA was performed with all samples compared to OVA, *p<0.05, **p<0.01,*

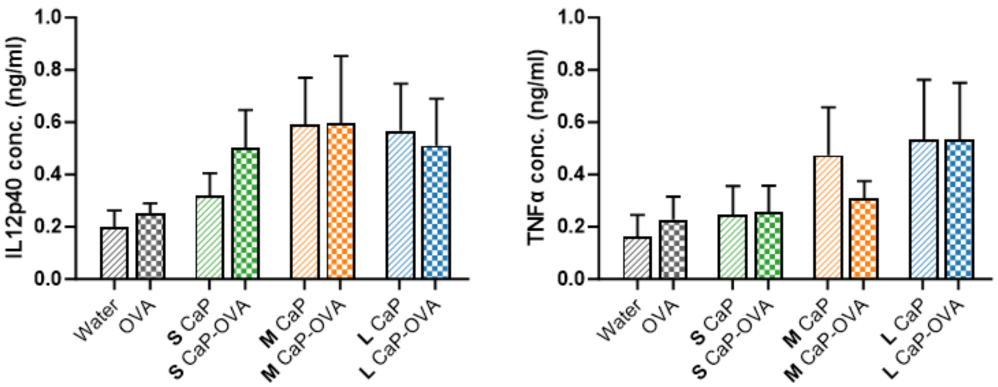

*Figure **S8**: IL-12p40, TNF-α cytokine quantification using ELISA from the supernatant of the DCs upon 18 hrs stimulation with different CaP NPs with and without OVA. Data is presented as the mean+SEM from 4 independent biological experiments.*
